# Deficiency of adenosine deaminase 2 (DADA2) with bilateral renal subcapsular hematoma: a case report and literature review

**DOI:** 10.1097/MS9.0000000000001812

**Published:** 2024-06-04

**Authors:** Anas R. Tuqan, Anas M. Barabrah, Basel A. Zaben, Mohammad Hakam Shehadeh, Motaz M. Adas

**Affiliations:** a Faculty of Medicine, Al-Quds University, Jerusalem; b Department of Internal Medicine, Palestine Medical Complex, Ramallah, Palestine

**Keywords:** adenosine deaminase 2, case report, DADA2, subcapsular hematoma

## Abstract

**Introduction and importance:**

Deficiency of adenosine deaminase 2 (DADA2) is a rare autosomal recessive genetic disorder caused by loss-of-function mutations in the adenosine deaminase 2 (ADA2) gene. This condition primarily manifests in pediatric cases before the age of 10 years, with sporadic cases reported in adults. ADA2 is a critical enzyme involved in macrophage differentiation and immune homeostasis. The clinical manifestations of DADA2 vary widely and can affect multiple organ systems. Our case uniquely highlights an infrequent DADA2 manifestation.

**Case presentation:**

An 18-year-old female presented with right flank pain, fever, and a history of joint pain, Raynaud’s phenomenon, livedo-like rash, and chronic abdominal pain. Physical examination revealed subcapsular hematoma in the right kidney. Further evaluation showed positive serologic tests for rheumatoid factor and antinuclear antibody (ANA). Genetic testing confirmed DADA2 homozygosity. The patient was discharged on the appropriate medications.

**Clinical discussion:**

DADA2 is associated with vascular dysfunction and systemic vasculopathy. The clinical manifestations of DADA2 encompass a spectrum of organ involvement, including the skin, nervous system, gastrointestinal system, renal system, and the cardiovascular system. Early recognition and diagnosis are crucial for appropriate management.

**Conclusion:**

This case report highlights the diverse clinical presentations of ADA2 deficiency, specifically focusing on bilateral renal subcapsular hematoma. This finding emphasizes the importance of considering DADA2 as a differential diagnosis in patients presenting with unexplained renal manifestations. Increased awareness of the varied clinical presentations of DADA2 will contribute to earlier diagnosis, appropriate management, and improved outcomes in patients affected by this rare genetic disorder.

## Introduction

Highlights

- Deficiency of adenosine deaminase 2 (DADA2) patients has high similarity to pan vasculitis manifestation.
- DADA2 vary widely and can affect multiple organ systems.
- Renal subcapsular hematomas are crucial complication of DADA2.

Deficiency of adenosine deaminase 2 (DADA2) is an autosomal recessive genetic disorder caused by loss-of-function mutations in the adenosine deaminase 2 (ADA2) gene (22q11.1)^1^. Originally identified in 2014 through independent research studies, this rare condition primarily manifests in pediatric cases before the age of 10, with sporadic instances reported in adults^2^. Approximately 200 cases have been documented since its recognition, suggesting an estimated prevalence of four cases per 100 000 individuals^1^.

ADA2 is an extracellular enzyme critical for macrophage differentiation, monocyte proliferation, and autocrine activity. ADA2 deficiency leads to impaired M2 macrophage differentiation, resulting in an elevation of proinflammatory M1 macrophages. These M1 macrophages release cytokines, triggering inflammation, causing damage to endothelial cells, and ultimately disrupting vessel wall integrity. The implications of ADA2 deficiency underscore its vital role in maintaining vascular health and immune homeostasis^1^.

The clinical manifestations of DADA2 encompass a spectrum from minor skin abnormalities to severe and potentially fatal organ involvement. Predominantly affecting the skin and central nervous system, this condition exhibits varying impacts on other tissues, including the gastrointestinal, liver, renal, and cardiovascular systems^1^. According to the recommendations put forth by the 2023 DADA2 Consensus Committee, the established diagnostic procedure involves either an enzymatic assay or genetic analysis. A diagnosis is confirmed when activity levels fall below 5%, as stipulated by these recommendations^3^.

This article presents a noteworthy case of ADA2 deficiency with bilateral renal subcapsular hematoma, where the patient initially presented with flank pain. The case underscores the importance of understanding and addressing the diverse clinical presentations associated with DADA2, contributing to the broader understanding of this rare genetic disorder.

This case has been reported in line with CARE criteria^4^.

### Case presentation

An 18-year-old single female came to the hospital on 15th October 2022 with a 10-days history of right flank pain. Pain was sudden in onset, radiating to the lower abdomen, and was associated with fever. There was no history of dysuria, nausea, vomiting, or trauma. She had a history of joint pain with morning stiffness lasting more than 60 min, Raynaud’s phenomenon and livedo-like rash with exposure to cold weather, chronic abdominal pain, and microscopic hematuria.

Her past medical history is significant for recurrent focal neurologic deficits with the last admission 3 years ago where brain MRI was done twice showing periventricular vasculitis, and then the patient lost follow-up. Family history is significant for a cousin with a deficiency of adenosine deaminase 2 polyarteritis nodosa (DAD2 PAN) syndrome, a father with rheumatoid arthritis, and a brother with an unknown vascular disease.

On general examination, the patient is oriented to time, place, and person but seems uncomfortable because of pain. Her vital signs were within normal limits except for fever which recorded about 38°C. On physical examination, the abdomen was soft and depressible, the chest was clear, the resting heart rate was normal, there was no lower limb edema, and there were no signs of joint inflammation or tenderness. However, left jugulodigastric lymphadenopathy measuring 1×2 cm, rubbery, tender, mobile, with overlying painful molar teeth was noticed. On further examination, positive tenderness was noticed on the right side of the abdomen along with costovertebral angle tenderness. An abdominal ultrasound revealed a subcapsular hematoma on the right kidney measuring ~10.5×6 cm, which compressed the renal parenchyma. Otherwise, both kidneys were normal in size, shape, echotexture, and position with no pronounced hydronephrosis. The patient was consequently admitted to the urology department with a right renal subcapsular hematoma.

**Figure 1:**
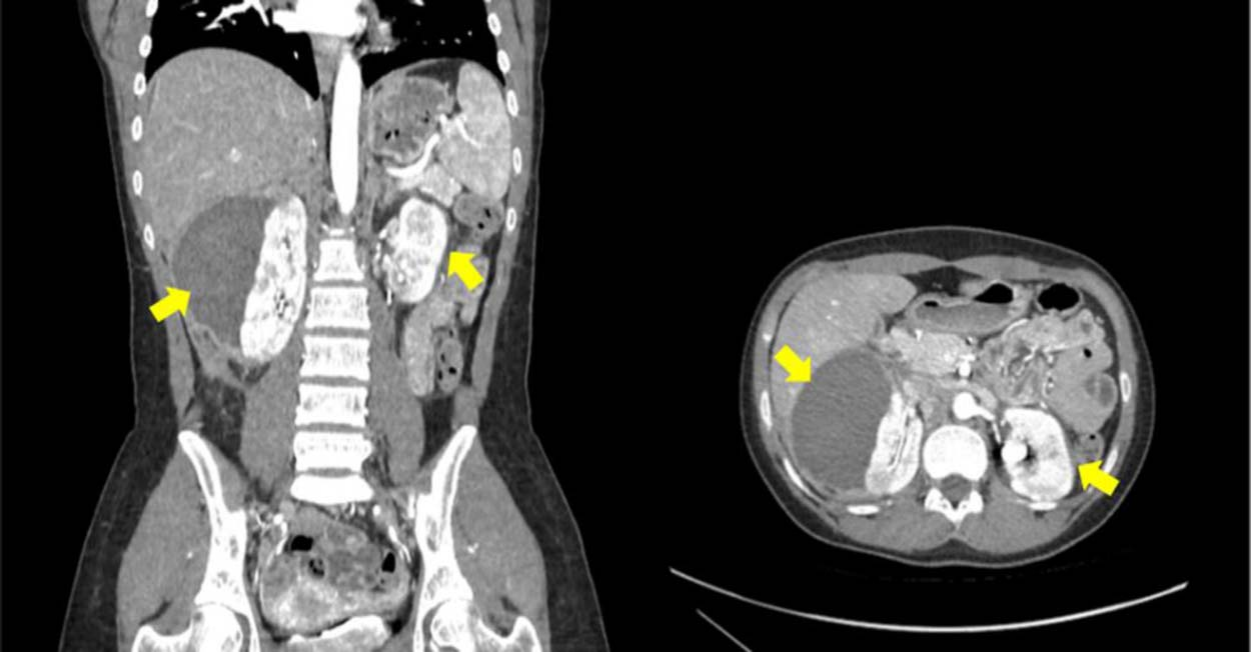
Computed tomography scan showing bilateral renal subcapsular hematoma, as well as multiple bilateral small renal aneurysms.

On 1st day of admission, the patient had the same complaints of the 1st presentation and a renal ultrasound was ordered. Ultrasound revealed that the previously seen right renal subcapsular hematoma had progressed in size and measured 11.4×5.7 cm with a noticeable increase in the vascularized hyperechoic component consisting of clot formation, as well as left sided subcapsular hematoma measuring about 2.6×0.6 cm. The patient after that was put on steroids and followed up. A contrast-enhanced CT scan revealed bilateral renal subcapsular hematoma that is more pronounced on the right measuring 7.5×5 cm and another small left subcapsular hematoma measuring 1.7×1 cm [Fig. 1].

Complete blood cell count and biochemical test results showed mild microcytic anemia [hemoglobin level: 11.6 g/dl, MCV: 72 μm^3^]. Urine analysis revealed protein +1, blood +3, and many RBCs. CRP and corrected ESR were recorded as 63 and 31, respectively. Serologic tests for rheumatoid factor and antinuclear antibody (ANA) were positive. The ENA profile was negative.

Noncontrast multiplanar multisequence MRI of the brain showed normal size and configuration of the ventricular system with no midline shift or mass effect except for nonspecific small scattered periventricular and deep white matter T2/FLAIR hyperintense foci. No evidence of acute intracranial abnormality noted such as infarction or hemorrhage.

Ultrasound on the 9th day of admission revealed that the right renal subcapsular hematoma had subsided slightly measuring 10.5×5 cm. The left kidney was otherwise normal, and no abnormalities were observed.

The first impression was PAN or PAN-like syndrome, which could be adenosine deaminase 2 deficiency (ADA2) or familial Mediterranean fever (FMF); therefore, she was given a referral report to perform a full vasculitis plan, as well as ADA2 and FMF gene mutations. The patient was then discharged on the 10^th^ day of admission on prednisolone.

**Figure 2:**
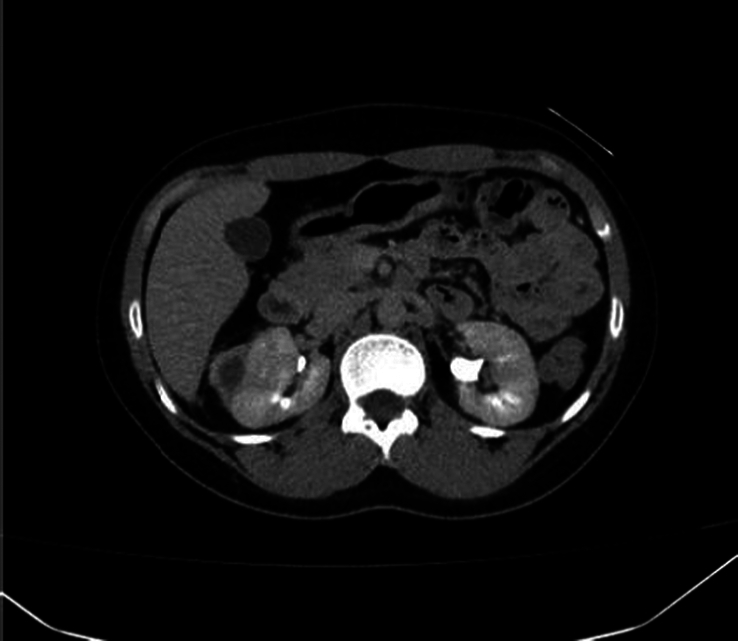
Computed tomography scan showing great regression in the subcapsular hematoma on the right kidney.

On evaluation of her visit at the renal clinic in another hospital, a CT scan was ordered on 13^th^ March, 2023 which revealed multiple small cortical defects in both kidneys, suggesting small infarction areas, associated with two small intralobular aneurysms, one at the right kidney and the other at the left. As for the previously seen subcapsular hematoma, it had become smaller in size measuring 4×3 cm. A small round aneurysm arising from branches of the gastroduodenal artery was also noticed, which appeared to be adjacent to the first part of the duodenum measuring approximately 4 mm [Fig. 2].

Renal US on 21st July, 2023 showed normal-sized kidneys with normal cortical thickness and no associated hydronephrosis. The patient is being followed up on a monthly basis by doing various blood tests (CBC, inflammatory markers, liver enzymes, and acute phase reactants), and she also takes etanercept once weekly as a subcutaneous injection as a maintenance therapy.

## Discussion

Deficiency of adenosine deaminase 2 (DADA2) is an autosomal recessive disorder resulting from loss-of-function mutations in the adenosine deaminase 2 gene (22q11.1), previously identified as CECR1 (cat eye syndrome chromosome region candidate 1)^1^. This rare condition was initially documented in 2014 through two distinct studies^2,5^. Rare instances of DADA2 individuals with adult onset have been documented, despite the condition being mostly seen in juvenile cases with symptoms appearing before the age of 10^6^. A prevalence of 4 per 100,000 people has been estimated based on the 200 cases that have been recorded since 2014^1^.

ADA2 is an extracellular enzyme that is mostly produced by monocytes and myeloid cells. It is a growth factor influencing the development of endothelial and hematopoietic cells. It causes macrophage differentiation, encourages monocyte proliferation, and has autocrine action. Deficiency of ADA2 results in impaired M2 macrophage differentiation, leading to elevated proinflammatory M1 macrophages that release cytokines, causing inflammation, endothelial cell damage, and vessel wall injury, ultimately disrupting vessel wall integrity. DADA2 is associated with clinical features of small or medium-sized vessel vasculitis, including lymphopenia, hypogammaglobulinemia, and systemic vasculopathy. Increased interferon (IFN) type I signatures suggest involvement of the IFN pathway. Studies in Japan have highlighted upregulated IFN type I and type II signaling pathways in DADA2, with the STAT1 gene implicated in pathogenesis. ADA2 mutation disrupts the enzyme’s conversion of adenosine and 2′-deoxyadenosine, leading to neutrophil activation, immune dysfunction, vasculopathy, and catalytic dysfunction, resulting in elevated adenosine levels, leading to inflammation, tissue damage, and fibrosis^1^.

The clinical manifestations of DADA2 might vary from minor skin abnormalities to serious, possibly fatal organ involvement. Skin and central nervous system involvement are predominant; other tissues that are impacted to varied degrees include the gastrointestinal, liver, renal, and cardiovascular systems. Fever and increased erythrocyte sedimentation rate or C-reactive protein levels are seen in about 50% of individuals. In a tiny percentage of cases, myalgia, arthralgia, and arthritis that particularly affects small joints are noted. Cutaneous manifestations are the most prevalent feature, present in over 75% of patients. Neurological events are experienced by 50% of individuals such as stroke, permanent polyneuropathy, cranial nerve palsy, central neuropathy, peripheral neuropathy, or transient ischemic attack. Vasculitis associated with DADA2 can extend its impact to organs such as the liver, kidney, and others. Several case series have documented arterial hypertension, renal artery stenosis, renal amyloidosis, renal artery aneurysm, kidney inflammation characterized by dense lymphocytic infiltration, and glomerular scarring. Up to 10% of patients have reported having gastrointestinal symptoms, such as inflammatory bowel disease and stomach discomfort. Additionally, some individuals may present with cytopenia as the initial manifestation or a concurrent feature of the disease^1,7^.

Diagnosing ADA2 deficiency poses a challenge due to its wide-ranging clinical presentation^8^. Nevertheless, specific signs such as early onset stroke, parental consanguinity, or a familial history of the condition, along with a lack of response to standard treatments, should prompt consideration for ADA2 deficiency^8,9^. As per the recommendations of the 2023 DADA2 Consensus Committee, the enzymatic assay or genetic analysis constitutes the established diagnostic protocol, with activity levels below 5% confirming the diagnosis^3^. The assessment of plasma ADA2 enzyme activity has exhibited high sensitivity and specificity in confirming DADA2^10^. Imaging techniques like MRI are more adept at identifying cerebral strokes compared to computed tomography (CT) scans^11^. In our case, the initial presentation of flank pain in our patient led to ultrasound and CT examinations, revealing bilateral renal subcapsular hematoma, initially suggesting a diagnosis resembling PAN or a PAN-like syndrome. Consequently, the plan included conducting a vasculitis work-up, in addition to evaluating DAD2 and FMF gene mutations. The genetic testing confirmed homozygosity for DAD2, thus validating the diagnosis of ADA2 deficiency in accordance with the established diagnostic criteria.

Distinguishing between DADA2 and other ailments sharing similar clinical presentations, like PAN, can present a considerable challenge^11^. As estimated in the literature, nearly 25% of DADA2 cases were incorrectly diagnosed as childhood PAN^11^. Although certain symptoms of DADA2 often mirror those of PAN, such as spontaneous perirenal hematoma or progressive chronic kidney disease, which make it hard to effectively distinguish between DADA2 and PAN^11,12^. Nonetheless, specific disparities do exist; DADA2 typically manifests at a younger age, exhibits unique skin manifestations such as livedo, and more frequently involves the central nervous system, resulting in ischemic strokes and brain hemorrhage^11,12^. Laboratory findings, encompassing reduced levels of immunoglobulins and platelets, also differ between DADA2 and PAN^11^.

TNF-α inhibitors, as The Consensus Committee states, stand as the primary treatment for DADA2, playing a major role in averting severe complications^11,12^. Complementary therapies encompass intravenous immunoglobulins (IVIG) for recurrent infections and neuroprotective measures during acute stroke episodes^12^. Moreover, Hematopoietic cell transplantation (HCT) exhibits promise in addressing the immunological, hematological, and vascular aspects of DADA2, yet its application comes with limitations and potential adverse effects^12,13^. In our patient’s case, positive responses were observed with Anti-TNF- α (etanercept) for symptom management, along with candesartan effectively controlling her blood pressure.

DADA2 is characterized by systemic vasculitis, early-onset strokes, bone marrow failure, and compromised immunity, affecting both children and adults. Its vasculitic nature leads to multiple organ complications, such as kidney issues like renal subcapsular hematomas and intralobular aneurysms^11–13^. Our reported case exhibited bilateral hematoma, managed in line with established consensus guidelines. Hematologic irregularities like pure red cell aplasia contribute significantly to anemia and cytopenia^13^. Also, there is a susceptibility to recurring infections posing substantial health risks due to compromised immune system^12,13^. These complications highlight the critical need for early intervention to manage stroke risks, vasculopathy, and immune deficiencies in ADA2 deficiency.

Our case reports a distinctive manifestation of ADA2, an infrequent complication, and emphasizes the necessity for utilization of an early diagnostic technique for identification. The constraints in our case arise from the scarcity of instances documented in the literature. Therefore, enhancing our understanding of the disease’s pathogenesis will aid in foreseeing clinical consequences, establishing more effective laboratory biomarkers, averting severe complications, and devising optimal treatment strategies tailored to the presentation of the disease.

## Conclusion

In conclusion, this case encourages increased awareness among healthcare professionals about the clinical spectrum of DADA2, contributing to earlier diagnosis, optimal management, and improved outcomes for patients with this rare genetic disorder. Further research is needed to better understand the pathophysiology of DADA2, diagnostic strategy, and to explore potential therapeutic approaches.

## Ethical approval

This study is exempt from ethical approval at our hospital.

## Consent

Written informed consent was obtained from the patient for publication of this case report and accompanying images. A copy of the written consent is available for review by the Editor-in-Chief of this journal on request.

## Source of funding

The study did not receive any funding.

## Author contribution

A.T., A.B., M.A.: data collection; A.T., A.B., B.Z., and M.H.S.: writing the manuscript; A.T., A.B.: study concept or design; A.T., A.B., and M.A.: review and editing the manuscript.

## Conflicts of interest disclosure

The authors declare no conflicts of interest.

## Research registration unique identification number (UIN)

Not applicable.

## Guarantor

Motaz M. Adas.

## Data availability statement

Not applicable.

## Provenance and peer review

Not commissioned, externally peer-reviewed.
